# Recurrent Invasive Pneumococcal Disease in Children: Underlying Clinical Conditions, and Immunological and Microbiological Characteristics

**DOI:** 10.1371/journal.pone.0118848

**Published:** 2015-03-04

**Authors:** Laia Alsina, Maria G. Basteiro, Hector D. de Paz, Melania Iñigo, Mariona F. de Sevilla, Miriam Triviño, Manel Juan, Carmen Muñoz-Almagro

**Affiliations:** 1 Allergy and Clinical Immunology Department, Hospital Sant Joan de Déu, University of Barcelona, Barcelona, Spain; 2 Department of Pediatrics, Hospital Sant Joan de Déu, University of Barcelona, Barcelona, Spain; 3 Department of Molecular Microbiology, Hospital Sant Joan de Déu, University of Barcelona, Barcelona, Spain; 4 Immunology Service, Hospital Clinic and Instituto de Investigaciones Biomédicas August Pi y Sunyer (IDIBAPS), Barcelona, Spain; 5 Functional Unit of Immunology, Hospital Sant Joan de Déu and Hospital Clinic. Barcelona, Spain; Centers for Disease Control & Prevention, UNITED STATES

## Abstract

**Purpose:**

Clinical, immunological and microbiological characteristics of recurrent invasive pneumococcal disease (IPD) in children were evaluated, differentiating relapse from reinfection, in order to identify specific risk factors for both conditions.

**Methods:**

All patients <18 years-old with recurrent IPD admitted to a tertiary-care pediatric center from January 2004 to December 2011 were evaluated. An episode of IPD was defined as the presence of clinical findings of infection together with isolation and/or pneumococcal DNA detection by Real-Time PCR in any sterile body fluid. Recurrent IPD was defined as 2 or more episodes in the same individual at least 1 month apart. Among recurrent IPD, we differentiated relapse (same pneumococcal isolate) from reinfection.

**Results:**

593 patients were diagnosed with IPD and 10 patients died. Among survivors, 23 episodes of recurrent IPD were identified in 10 patients (1.7%). Meningitis was the most frequent form of recurrent IPD (10 episodes/4 children) followed by recurrent empyema (8 episodes/4 children). Three patients with recurrent empyema caused by the same pneumococcal clone ST306 were considered relapses and showed high bacterial load in their first episode. In contrast, all other episodes of recurrent IPD were considered reinfections. Overall, the rate of relapse of IPD was 0.5% and the rate of reinfection 1.2%. Five out of 7 patients with reinfection had an underlying risk factor: cerebrospinal fluid leak (n = 3), chemotherapy treatment (n = 1) and a homozygous mutation in MyD88 gene (n = 1). No predisposing risk factors were found in the remainder.

**Conclusions:**

recurrent IPD in children is a rare condition associated with an identifiable risk factor in case of reinfection in almost 80% of cases. In contrast, recurrent IPD with pleuropneumonia is usually a relapse of infection.

## Introduction

*Streptococcus pneumoniae* (pneumococcus) is a commensal organism of the human nasopharynx [1] and a major cause of morbidity and mortality worldwide. Although all age groups may be affected, the highest rate of pneumococcal disease occurs in young children and in the elderly [2]. In 2000, it was estimated that about 14.5 million episodes of serious pneumococcal disease and more than 800,000 deaths in children less than 5 years of age occurred [3]. In addition, pneumococcus is the leading cause of mild infections of the upper respiratory tract (otitis media, sinusitis) [1]. Despite this high burden, the occurrence of 2 or more episodes of invasive pneumococcal disease (IPD) in the same individual one month apart, called recurrent IPD [4], is much less frequent, with an estimated rate between 2 and 4% [4]. The event of a recurrent IPD must then pose the question of an underlying medical condition predisposing to this rare phenomenon.

The best-known acquired factors that determine susceptibility to IPD, other than young age, are co-infection by the human immunodeficiency virus (HIV), splenectomy, malignancies, chronic cardiopulmonary diseases, traumatic cerebrospinal fluid leaks and cochlear implant [5]. Inherited factors have also long been known to predispose patients to IPD, notably sickle-cell disease and primary immunodeficiency diseases (PIDs). Classically, the PIDs most related to IPD were congenital asplenia, defects in the classical pathway of complement activation and defect in the antibody response to polysaccharides [6]. All these PIDs share a common pathophysiological mechanism, which is a high level of interference with the ability of opsonization and phagocytosis of the encapsulated bacteria by the splenic macrophages. Patients suffering from these PIDs are not only prone to IPD, but also to serious infections by other encapsulated bacteria (*Streptococcus* group B, *Haemophilus influenzae* type B, *Salmonella typhi*, *Neisseria meningitidis*). Recently a new group of PIDs was identified, affecting innate immunity and predisposing to infection by Gram-positive bacteria, pneumococcus in particular [7–12]. They include anhidrotic ectodermal dysplasia with immunodeficiency [8] [of which there are two forms, X-linked and autosomal dominant, caused by defects in *NF-kappa-B essential modulator* (NEMO) or gain-of-function in *alpha inhibitor ofNF-kappa-B* (IKBA)], and the IL-1 receptor-associated kinase type 4 (IRAK-4) deficiency [7–12] and myeloid differentiation primary response 88 (MyD88) deficiency [11–12], both autosomal recessive. These are monogenic diseases in which there is a mutation in the NEMO gene, IRAK-4 or MyD88, respectively, whose products are crucial in the signaling pathways of Toll-like receptors (TLR) and IL-1 receptors (the Toll-IL1R or TIR pathway), the most relevant sensors of pathogens and inflammation in the innate immune system [13].

In recent years there have appeared several publications analyzing the underlying factors in IPD, but only a few have focused on patients with recurrent IPD in children [4,14,15]. Their results demonstrated that underlying risk conditions were present in 80% of patients with recurrent IPD, both children and adults, and which might not have been identified before the IPD recurrence [15]. Of note, most patients with recurrent IPD without underlying risk conditions were young children less than 12 months old [4].

The aim of this study was to evaluate the clinical features of a pediatric population displaying recurrent IPD, as well as to identify the underlying microbiological characteristics and risk factors associated with this condition. We performed a broad immunological evaluation of the patients with no apparent underlying factors, including evaluation of the TIR pathway that has only been performed in one recent study [16].

## Patients and Methods

### Patients and Setting

We identified all children and adolescents with IPD who were admitted to Sant Joan de Déu Hospital in Barcelona (January 2004 to December 2011). Our hospital is located in the southern area of Barcelona, Catalonia, Spain, and serves a paediatric referral population of 200,000 children <18 years, around 19% of the Catalan paediatric population (data obtained from Catalonian Department of Statistics, www.idescat.net). Conjugate vaccines are not subsidized by the Spanish Health Service but the uptake of the first one available, heptavalent conjugate vaccine (PCV7), has increased since its introduction in June 2001. It has been estimated to have provided PCV7 coverage of around 50% in the year 2009 [17]. In 2010 PCV7 was replaced by high-valent conjugate vaccines (PCV10 and PCV13).

An episode of IPD was defined as the presence of clinical findings of infection together with isolation and/or DNA detection of *pneumolysine (ply)* gene and an additional capsular gene (*wzg*) of *S. pneumoniae* by Real-Time PCR in any sterile body fluid such as blood, cerebrospinal fluid, pleural fluid or articular fluid. A recurrent IPD was defined as 2 or more episodes of IPD in a same individual at least one month apart [17]. Among recurrent IPDs, we differentiated relapse IPD if the same pneumococcal isolate was identified (defined as 2 strains with the same clonal type and/or serotype) and reinfection IPD if it was different (defined as 2 strains with different serotype and/or clonal type).

The Clinical Microbiology Laboratory monitored all confirmed pneumococcal infections and several variables were routinely recorded including demographic data, identification hospital number, type of infection, antimicrobial susceptibility, serotype and clonal type or sequence type. IPD was classified according to the International Classification of Disease Ninth Revision (ICD-9) specific for diseases caused by *S. pneumoniae* as follows: meningitis, pneumonia, parapneumonic empyema, occult bacteremia, sepsis, arthritis, peritonitis, and endophthalmitis. Identification number of all isolates from sterile fluids was used to review electronic medical records and recorded demographic, epidemiological and clinical variables including age, gender, date of birth, date of admission, clinical data, treatment, outcome, and pneumococcal polysaccharide-protein conjugate vaccination status. A thorough medical chart review and interview with patients with recurrent IPD and family was performed in order to identify underlying medical conditions that are known to predispose to IPD [5]. Brain magnetic resonance imaging (MRI) was performed in patients who experienced one or more episodes of meningitis, to rule out CSF leak.

### Immunological studies

In the event that no risk factor was identified (HIV, splenectomy, malignancies, chronic cardiopulmonary diseases, traumatic cerebrospinal fluid leaks, cochlear implant, sickle-cell disease, drug-induced immunosuppression), the patient was referred to the clinical immunologist in our hospital for immune evaluation, which included, stepwise, the following tests: 1) evaluation of specific antibody responses: immunoglobulin levels (IgG, IgA, IgM), IgG subclass levels, T/B/NK lymphocyte phenotyping, antibodies against polysaccharide antigens (isohemagglutinins and pneumococcus, post-infection), and protein antigens (diphtheria, tetanus, pneumococcus post-Prevenar vaccination); 2) evaluation of innate immunity: CH50 (assay for evaluation of classical pathway by 50% of hemolysis), C3 and C4 levels, Howell-Jolly bodies in blood smear and/or abdominal ultrasound (both to rule out asplenia), and evaluation of TIR pathway through CD62L shedding [18] and whole blood stimulation with TLR ligands and cytokine measurement (defined in the paragraph that follows) [7–11]. In patients suffering from pneumonia or parapneumonic pleural effusion as the only form of recurrent IPD, with no other infections, the TIR pathway was not evaluated, since isolated pneumonia is not a common presentation of TIR deficiencies [18,19].

IgG, A and M and complement C3, C4 were evaluated using immunoturbidimetric specific protein methods (Architect ci8200). CH50 was determined by ELISA (Binding Site). IgG subclasses were evaluated by nephelometric technique. Specific polysaccharide antibody deficiency was defined as normal serum IgG, A and M and IgG subclass levels and a defect of the antibody responses to *S. pneumoniae* (or other polysaccharide vaccine) either after documented invasive infection or after test immunization and/or absent isohemagglutinins above 1 year of age.

Immunodeficiences were defined according criteria of ESID (European Society Immunodeficiences). http://esid.org/Working-Parties/Registry/Diagnosis-criteria. IgA + IgG subclass deficiencies were defined in patients aged>4years with marked decrease in IgA [ie <0.05g/l] and at least one of IgG 1–3 subclasses less than the 5th percentile for age along with poor response to vaccines and/or negative isohemagglutinins (<1/8). IgG subclass deficiencies were defined in patients aged>4years with normal levels of IgM and IgA at least two of IgG 1–3 subclasses less than the 5th percentile for age along with poor response to vaccines and/or negative isohemaglutinins (<1/8). Specific polysaccharide deficiency was defined in patients >4 years with normal levels of IgG subclasses and IgA, and poor polysaccharide response (negative isohemaglutinins and/or absent pneumococcal antibodies after natural infection or less than 2-fold increase in pneumococcal antibodies after polysaccharide vaccination with Pneumovax23). Hypogammaglobulinemia was defined as marked decrease of at least one of Ig,IgG subclass(es), IgA or IgM levels (measured at least twice), secondary causes of hypogammaglobulinaemia having been excluded, with normal isohaemagglutinins and/or antibody response to vaccines. Complement deficiencies required genetic confirmation of the defect after detection of low CH50 in two different determinations 6 months apart.

### Evaluation of TIR pathway of whole blood (WB) stimulation with TLR ligands through CD62L shedding and cytokine measurement [7–11]

WB was diluted with an equal amount of RPMI before adding different stimuli. Diluted WB was activated for 24 hours with 10μg/ml of polymyxin B to clear LPS contamination plus TLR1/2 agonist Pam3CSK4 0.5μg/ml (Invivogen, Cayla, France); TLR2/6 agonist FSL1 1μg/ml (Invivogen); TLR4 agonist LPS 100ng/ml (Invivogen); 1μg/ml TLR5 agonist Flagellin-BS (Invivogen); 1μg/ml TLR7/8 agonist Imiquimod (Invivogen); TNFα 0.2μg/ml (Miltenyi Biotech, Bergisch Gladbach, Germany); PMA 2μg/ml (Sigma Aldrich, St Louis, MO, USA) or left unstimulated, at 37°C and 5% CO2. The expression of CD62L on neutrophils was measured by flow cytometry (FACScalibur, BD Bioscience, NJ, USA), at baseline and after 1 h TLR stimulation as published before [20]. Cytokine production (GM-CSF, IFN-γ, IL-1β, IL-2, IL-4, IL-5, IL-6, IL-8, IL-10 and TNF-α) was measured at 24 h after stimulation through Luminex assay (LifeTechnologies, Carlsbad, NM, USA).

### Microbiological studies

All pneumococcal isolates were identified by standard microbiological methods that did not change during the study period. Agar dilution technique was used to determine the MIC (defined as the lowest concentration of an antibiotic needed to inhibit visible bacterial growth) of several antibiotics, including penicillin and cefotaxime. American Type Culture Collection (ATCC) 49619 (serotype 19) was used as a control. Antibiotic susceptibilities were defined according to the 2013 breakpoints of the European Committee on Antimicrobial Susceptibility Testing (EUCAST) http://www.eucast.org/clinical_breakpoints/

Isolates with resistance to 3 or more antimicrobial classes were considered multiresistant.

Detection of *ply* gene of *S. pneumoniae* was performed with quantitative Real-Time PCR according to a published assay [21]. Serotyping of strains isolated by culture was carried out with the Quellung reaction, using antisera provided by the Statens Serum Institut (Copenhagen, Denmark), or with Dot-Blot serotyping. MICs and serotyping of the strains were performed at the National Center for Microbiology (Majadahonda, Madrid). Detection of pneumococcal serotypes in negative culture clinical samples that were *ply* pneumococcal gene positive was performed according to a published Multiplex Real-Time PCR methodology [22]. This procedure includes the DNA detection of conserved *wzg* capsular gene of *S. pneumoniae* and other genes selected to distinguish 24 serotypes (1, 3, 4, 5, 6A, 6B, 7F ⁄ A, 8, 9V ⁄ A ⁄ N ⁄ L, 14, 15B ⁄ C, 18C ⁄ B, 19A, 19F ⁄ B ⁄ C, 23A, and 23F). Clonal type of strains was analyzed using Multi-Locus Sequence Typing (MLST) as reported elsewhere [23]. The assignment of alleles and sequence types (ST) was carried out using the software at the pneumococcal web page www.mlst.net

### Statistical analysis

We used the χ^2^ test or Fisher’s exact test to compare proportions, and student’s t-test to compare means. Statistical analyses were performed using SPSS for Windows, version 17.0 (SPSS), and Epi Info, version 6.0 (Centers for Disease Control and Prevention). We calculated 95% CIs, and 2-sided *P* values ≤0.05 were considered to be statistically significant.

### Ethical issues

Written informed consent was obtained from parents or legal guardians of patients included in the study. Data and informed consent were recorded following the guidelines of the Sant Joan de Deu Hospital Clinical Research Ethical Committee, which approved the study.

## Results

During the study period a total of 606 episodes (588 hospital admission episodes) of IPD occurred in 593 patients. Three-hundred sixty-three episodes were identified only by positive detection of pneumococcal DNA by Real-Time PCR with negative bacterial culture. Among 593 patients, bacteremic pneumonia occurred most frequently in the first episode of this cohort (n = 439; 74%, 311 of them with empyema), followed by bacteremia/sepsis (n = 75; 13%), meningitis (n = 64; 11%), arthritis (n = 12; 2%), appendicitis (n = 2) and endophthalmitis (n = 1). The median age of patients was 36 months, range 18 days-18 years, and they included 339 males (57.2%) and 254 females (42.8%); 10 patients (7 males and age range 4 months-15 years) died, yielding a mortality rate of 1.7%. Metabolic disease (n = 3) cerebral palsy (n = 1) and lymphoblastic leukaemia (n = 1) were identified as underlying risk factor in 5 of these 10 patients. No risk factors were identified in the other patients despite two of them having no appropriate elevation of C-reactive protein during the acute-phase of infection.

### Rate and type of recurrent IPD

Among 583 children who survived the first episode of IPD, 10 (1.7%) had more than 1 episode with criteria of recurrent IPD: 8 children experienced 2 infections, 1 child experienced 3 infections and 1 child experienced 4 infections (n = 23 episodes). Sixteen out of 23 episodes (69.5%) were caused by serotypes included in the 13-valent conjugate vaccine. The most frequently detected serotypes were 1 (6/23, 26.1%), 6A (3/23, 13%), 6C (1/23, 4.3%) and 19A (3, 13.0%). Six out of 23 episodes (26%) were identified only by real-Time PCR, and antimicrobial susceptibility and sequence type were not available. Among the 17 strains, 15 different clonal types or sequence types were identified and 4 of them were multiresistant strains. Tables 1 and 2 show the main clinical and microbiological characteristics of children with recurrent IPD.

**Table 1:** Clinical characteristics of patients with recurrent invasive pneumococcal disease.

P	Type of recurrent IPD	Date of admission	Age (months)	Time to first episode (days)	Sex	Risk factors	Type of Pneumococcal vaccine	Diagnosis
1	Reinfection	19/02/2007	141	0	F	CSF leak	No	Meningitis
		02/01/2009	163	683			PPV-23	Meningitis
		19/12/2009	175	973				Meningitis
2	Reinfection	23/11/2006	60	0	M	CSF leak	PCV-7	Meningitis
		07/10/2007	69	211			PPV-23	Meningitis
		24/04/2008	75	378				Meningitis
		13/01/2009	84	464				Meningitis
3	Reinfection	14/02/2011	71	0	M	CSF leak	No	Meningitis
		26/12/2011	81	315			PPV-23	Meningitis
4	Reinfection	20/03/2006	25	0	F	Immunodeficiency	No	Arthritis
		13/08/2006	30	146		MyD88	PCV-7	Arthritis
5	Reinfection	02/12/2010	39	0	F	Chemotherapy	No	Bacteremia
		13/10/2011	49	315			No	Bacteremia
6	Reinfection	27/06/2011	14	0	M	No	No	Meningitis
		19/10/2011	17	114			PCV-13	Bacteremia
7	Reinfection	25/10/2009	27	0	F	No	PCV-7	Pneumonia
		07/01/2011	41	439		Flu H1N1	No	Empyema
8	Relapse	13/02/2007	68	0	M	No	No	Empyema
		28/03/2007	70	43			PPV-23	Empyema
9	Relapse	16/03/2007	76	0	F	No	PCV-7	Empyema
		24/04/2007	77	39			No	Empyema
10	Relapse	12/03/2007	134	0	F	No	No	Empyema
		12/04/2007	135	31			No	Empyema

P, patient identification; PCV-7, 7-valent polysaccharide-protein conjugate vaccine; PCV 13, 13-valent polysaccharide-protein conjugate vaccine; PPV-23, 23-valent pneumococcal polysaccharide vaccine.

**Table 2:** Microbiological characteristics of patients with recurrent invasive pneumococcal disease.

					MIC					DNA Pneumococcal load (copies/mL) Log 10			
P	Diagnosis	Serotype	ST	MDR	Ctx	Pen	Ery	Cm	tetra	Plasma	CSF	Pleural fluid	Other invasive non-pneumococcal infections
1	Meningitis	19A	416	-	0.015	0.015	0.12	4	0.25	3.8	7.8	NA	
	Meningitis	19A	199	-	0.03	0.015	0.12	4	0.25	NA	NA	NA	
	Meningitis	6C	4310	+	0.06	0.06	**128**	4	**64**	NA	7.0	NA	
2	Meningitis	13	2592	-	0.03	0.03	0.12	4	0.25	3.7	7.7	NA	Meningitis *H*.*influenzae*
	Meningitis	16F	30	+	0.12	0.03	**128**	**32**	**32**	NA	NA	NA	
	Meningitis	19A	276	+	1	**1**	**128**	4	**32**	NA	NA	NA	
	Meningitis	6A	1692	-	0.015	0.015	0.12	4	0.25	3.1	7.1	NA	
3	Meningitis	15A	63	+	0.12	**0.12**	**128**	4	**64**	NA	5.4	NA	
	Meningitis	10A	97	-	0.015	0.015	0.12	4	0.25	NA	NA	NA	
4	Arthritis	NA	NA^a^		NA	NA	NA	NA	NA	NA	NA	NA	
	Arthritis	19A	3438	-	0.12	**0.5**	0.12	4	0.25	NA	NA	NA	
5	Bacteremia	7F	1589	-	0.015	0.015	0.12	4	0.25	NA	NA	NA	
	Bacteremia	19F	5194	+	0.25	**0.5**	**128**	4	**64**	NA	NA	NA	
6	Meningitis	6A	490	+	0.015	0.015	**128**	4	**4**	NA	>8	NA	Meningococcal meningitis UTI (*Proteus*-BLEA)
	Bacteremia	6A	2611	-	0.015	0.015	0.12	4	0.25	NA	NA	NA	
7	Pneumonia	others	na^a^		NA	NA	NA	NA	NA	6.5	NA	NA	
	Empyema	5	1223	-	0.015	0.015	0.12	4	0.25	4.7	NA	7.4	
8	Empyema	1	306	-	0.015	0.03	0.12	4	0.25	2.9	NA	>8	
	Empyema	1	NA^a^		NA	NA	NA	NA	NA	NA	NA	6.8	
9	Empyema	1	306	-	0.015	0.015	0.12	4	0.25	4.4	NA	>8	
	Empyema	1	NA^a^		NA	NA	NA	NA	NA	3.4	NA	6.9	
10	Empyema	1	306	-	0.015	0.015	0.12	4	0.25	NA	NA	>8	
	Empyema	1	NA^a^		NA	NA	NA	NA	NA	NA	NA	6.8	

P, patient identification; ST, sequence type by Multi-locus sequence typing; MDR, Multi-drug resistant strain; PCV-7, 7-valent polysaccharide-protein conjugate vaccine; PCV 13, 13-valent polysaccharide-protein conjugate vaccine; PPV-23, 23-valent pneumococcal polysaccharide vaccine; CSF, cerebrospinal fluid; NA, Not available; MIC, minimal inhibitory concentration; Pen, penicillin; Ery, erythromycin; Cm, chloramphenicol; Tet, tetracycline; Ctx, Cefotaxime; Bold text, resistant strain according to MICs for *Streptococcus pneumonia*. EUCAST Clinical Meningeal Breakpoints.

^a^NA, ST were not available in episodes identified only by PCR.

None of the children who suffered recurrent IPD died during the study period. In terms of age, recurrent IPD was detected in 0.9% of patients younger than 5 years (4 of 444 children) and in 4.3% of children ≥5 years (6 of 139) P = 0.01.

Meningitis was the most frequent form of recurrent IPD, with 10 episodes (43.5%) in 4 children, followed by pulmonary infections, with 8 episodes (34.8%) in 4 children. Seven out of 8 episodes were pneumonia complicated with empyema, and one episode of non-complicated pneumonia. Other infections were occult bacteremia (3 episodes; 13.0%) and arthritis (2 episodes; 8.7%).

Three patients (2 episodes each) with recurrent empyema caused by *S*.*pneumoniae* ST306 were considered as having a relapse of infection. Of note, these 3 patients had an extremely high bacterial load (>10,000,000 copies/ml) in their first episode of empyema. The other episodes (n = 17) of recurrent IPD suffered by the remaining 7 patients were considered reinfection. Different serotypes and clonal types were detected in each child, except in two of them in whom the same serotype but different clonal type was found. The mean of days between episodes was 39 in relapses, and 314 in reinfection P <0.02. Overall, the rate of relapse of IPD was 0.5% and the rate of reinfection 1.2%. No significant differences were found in the rate of reinfection in children younger than 5 years (0.9%; 4 of 444) and ≥5 years (2.1%; 3 of 139) p = 0.2.

### Risk factors and immunological characteristics in children with recurrent IPD

Among the 10 patients with recurrent IPD, 4 patients had a well-recognized underlying risk factor: Cerebrospinal fluid (CSF) leak (3 patients) and chemotherapy treatment (1 patient). Five of the remaining 6 patients were then evaluated for immunodeficiency (Table 3).

**Table 3:** Immunological work-up of patients with recurrent invasive pneumococcal disease with unknown underlying risk factors.

							Ab levels				TolI-IL 1R evaluation	
P	Diagnosis	Howell-Jolly bodies^a^	IgG, IgA, IgM Levels^b^	IgG subclass levels^b^	LIP	Isohem-agglutinins^b^	Diptheria^c^	Tetanus^c^	Pneumo^c^	Complement pathway^d^	Shedding CD62L	Cytokine after stimulation
4	Arthritis	No/normal spleen	Normal	Normal	Normal	NA	Protective	Protective	Protective	Normal	Defective	Defective
6	MeningitisBacteremia	No/normal spleen	Normal	Normal	Normal	Anti-B 1/16	Protective	Protective	Protective	Normal	Normal	Normal
7	PneumoniaEmpyema	No/normal spleen	Normal	Normal	Normal	Anti-A 1/32	Protective	Protective	Protective	Normal	ND	ND
8	EmpyemaEmpyema	No/normal spleen	Normal	Normal	Normal	Anti-B 1/32	Protective	Protective	Protective	Normal	ND	ND
9	EmpyemaEmpyema	No/normal spleen	Normal	Normal	Normal	NA	NA	Protective	NA	Normal	ND	ND

P, Patient identification; LIP, Lymphocyte immune-phenotype (T/B/NK); NA, Not available; ND, Not determined; Data for patient 10 were not available.

^a^ In blood smear and/or abdominal ultrasound study.

^b^ Range for age.

^c^ Criteria of positive isohemagglutinins: antiA > 1/16, antiB > 1/8; Criteria of positive antibody (ab) levels of diphtheria and tetanus: > 0.1 UI/mL. Protective antibody (ab) levels > 250 UI/mL. Measurement of antibodies to 23 pneumococcal capsular polysaccharides through enzyme-linked immunosorbent assay.

^d^ Complement pathway includes CH50 (normal between 42–95 UI/mL), C3, C4 for all patients. AH50, properdin and MBL for patient 6.

For patient 4, a severe defect in the Toll-IL1R pathway was detected: a nearly absent CD62L shedding was observed on granulocytes upon activation with agonists of TLRs 1/2, 2/6, 5, 7/8. These results were confirmed with evaluation of cytokine production (GM-CSF, IFN-γ, IL-1β, IL-2, IL-4, IL-5, IL-6, IL-8, IL-10 and TNF-α) after 24h of whole blood stimulation with the same TLR agonists; this was also severely impaired (data not shown). A homozygous mutation in MyD88 gene (E65del/E65del) was identified, thus confirming the primary immune defect of the TIR pathway [11].

For patient 6, the first episode of IPD was meningitis and the second an occult bacteremia, 16 weeks later with the same serotype but different clonal type. The child had previously suffered meningococcal meningitis by serotype B. However, all the immune work-up turned out to be normal. Moreover, we detected an appropriate antibody response to pneumococcus after the first IPD episode, highlighting the absence of a defect in humoral immunity to polysaccharide antigens, such as pneumococcus capsule. Due to the association of pneumococcal and meningococcal infection in the patient, mannose binding lectin genotype, AH50 and Properdin levels were also determined; all were normal. After the second episode of IPD, and despite the normality of the immune work-up, the child was given amoxicillin prophylaxis, for suspected innate immune deficiency. No new episodes of invasive bacterial disease were recorded in the subsequent 2 years.

Among the remaining 4 children, no underlying recognized condition was detected, except coinfection with pandemic flu H1N1 in the second episode of patient 7. Of note, the type of recurrent IPD of all these patients was respiratory tract IPD, and in 3 of them, the recurrent IPD was identified as a relapse of empyema episodes.

### Vaccination status

Only 3 children had received at least one dose of PCV7 vaccine before the first IPD episode, and all of their IPDs were caused by non-PCV7 serotypes. After the first IPD episode, 5 out of 10 patients received at least one dose of another pneumococcal vaccine: four PPV23 and one PCV13. Three out of 4 patients who received PPV23 vaccine after the first episode had a new infectious episode caused by serotypes included in this vaccine (1, 10A and 19A). CSF leak was detected in all of them. Also, the only patient who had received 2 doses of the PCV13 after the first episode suffered a reinfection caused by a serotype included in PCV13 (serotype 6A). In this patient no risk factors were detected.

## Discussion

The present work aims at analyzing the underlying medical conditions and microbiological data of children with recurrent IPD in Barcelona, among a representative population in the north of Spain. There are two main conclusions that may be drawn from the results.

First, as expected, the frequency of recurrent IPD in this pediatric population is low (1.7%), even lower than other reported studies [4, 15, 16, 24]. It is well known that the rate of recurrence in children is lower than in adults [4, 25–27]. No patient died of recurrent IPD during the study period, which is in agreement with previous reports comparing IPD in children and adult population [24, 27].

Second, except for recurrent complicated pneumonia, all except one child with recurrent IPD had an underlying condition that should be identified by the practitioner in order to reduce future recurrences and related sequelae. In our study, the most frequent underlying disease was a local anatomical factor (CSF leak in recurrent meningitis). Indeed, meningitis was the most prevalent form of recurrence, and more than 2 episodes occurred despite vaccination. Thus, in cases of recurrent meningitis, there is a clear need to perform CNS imaging for the diagnosis of CSF leak. These data contrast with those reported by Gaschinard et al [16] and Einarsdóttir *et al*. [24], who described immunoglobulin disorders as the main risk factor for recurrent IPD in children. The levels of immunoglobulins IgG, IgA and IgM were determined in all patients with recurrent meningitis before the identification of the CSF leak and in all patients with no identified risk factor, and these levels were normal. In our population both primary and secondary immunodeficiencies were detected in 2 patients (and suspected in another): one patient was receiving chemotherapy and the other suffered an identified PID (MyD88 deficiency). The incidence of PID among patients with IPD might be higher than identified here where only recurrent IPD was studied. Indeed, 10 children died of IPD during the study period and 5 of them had underlying risk factors. Of note, 2 patients who died without underlying risk factors showed no C-reactive elevation that might be suggestive of a defect in the TIR pathway [19].

Our low rate of PID in children compared with that reported for Gashingnard et al [16] could be explained by the distribution of circulating serotypes in our geographical area. Temporal trends of serotypes causing IPD could be related with associated risk factors for IPD and for recurrent IPD. There are more than 94 pneumococcal serotypes that cause varying rates of IPD. Some of these serotypes are called “opportunistic serotypes”; they are frequently detected in carriers and they are more prevalent causing IPD in patients with comorbidities [28]. In contrast, serotypes with “high-attack-rate”, also called “high-invasive potential serotypes”, are seldom detected in carriers and often cause IPD particularly in older children and adults without comorbidities [28, 29]. Moreover, we recently reported that young children with genetically-determined low-mannose binding lectine (MBL) production are at a higher risk of developing IPD, particularly that caused by opportunistic or low-attack-rate pneumococcal serotypes [30]. Serotype 1, a well-known high attack serotype, was highly prevalent during the study period mainly associated to pneumonia [17,29], so this could explain a high proportion of disease in healthy children and consequently a low rate of recurrent IPD related with immunological disorders. Of note, in the study of Gaschingnard et al bacteremic pneumonia was not included as IPD. However, if bacteremic pneumonia episodes were not consider, the recurrent IPD of our study raise to 3,2%, that is still significantly lower in comparison with the 10% of Gaschingnard’s study.

Based on our results, recurrent pneumococcal pneumonia seems to be pathophysiologically different from other forms of recurrent IPD. Recurrent pneumonia would be more related to microbiological factors than to host factors. The three patients suffering complicated pneumonia without underlying predisposing conditions corresponded to episodes of relapse caused by the virulent clone ST306 expressing serotype 1. The interval between relapses was much lower (39 days) than between episodes of reinfection (314 days). Also, a much higher bacterial load in pleural fluid was detected despite the antibiotic course usually being standardized in the same way. This suggests that the main cause of this recurrence is insufficient treatment or clearance of the infection in the first episode of empyema. One limitation of our study was the inability to determine the clonal composition of strains detected in the second episode of the 3 cases of relapses detected in patients suffering complicated pneumonia. However, the high homogeneity of serotype 1 as previously described [29, 31] and the short time interval between episodes suggests that these were relapses rather than reinfections.

Another relevant aspect is the vaccination status of the patients at the time of pneumococcal infection. As expected, approximately 4.3% and 70% of identified serotypes were included in the PCV7 and PCV13 vaccines, respectively, reflecting the distribution of serotypes in our geographic area [31]. However, only 3 patients had received pneumococcal vaccine before the first episode of IPD (all PCV7), and 6 received vaccination afterwards (4 PPV23, 1 PCV13 and 1 PCV7). Surprisingly, the patient who suffered reinfection without an identified underlying condition presented a second episode of IPD caused by serotype 6A, which is included in the 13-valent vaccine, despite having received 2 doses of this vaccine between the two episodes and having achieved protective levels of pneumococcal antibodies (Ab). This discrepancy might be explained by the limitation in evaluating Ab responses to pneumococcus through the pool of 23 serotypes (ELIZEN Pneumoccocus IgG assay, ZenTech) instead of the quantification of the 13-serotype-specific Ab. As previous studies have shown, nonfunctional antibodies to the cell wall polysaccharides can interfere with the determination of serotype-specific antibodies in these ELISAs. In addition, because of the variable immunogenicity among different serotypes, the measurement of overall antibody responses can give rise to the observation of high levels of antibodies all induced by a single serotype, and may mask a possible deficiency in antibody responses to serotypes that are less immunogenic [32, 33].

Finally, a PID was diagnosed in a patient due to the broad immune work-up established in our hospital for recurrent IPD. This evaluation must include specific antibody responses to polysaccharides, complement and spleen function, since these three deficiencies are the most commonly described [16, 34]. The recommendation of including TIR evaluation in the immune evaluation of recurrent IPD in children was made in 2007 [35]. Recently, Gaschinard *et al* [16] published a systematic evaluation of PID, including TIR pathway, in a prospective study on 163 children hospitalized in France for IPD, of which 10% were recurrent. They identified 11% of patients with PID, with primary antibody and complement deficiencies being the most common. They also identified a patient with MyD88 deficiency after a single episode of pneumococcal meningitis and a previous episode of ethmoiditis. The identification of MyD88 deficiency in our patient enabled us to optimize her treatment and antibiotic prophylaxis, with an improved outcome. PIDs affecting TIR pathway are rare and represent less than 1% of all forms of PIDs [19], but due to their recent description, and to the need for performing highly specific tests for their diagnosis [11, 20], they are probably underdiagnosed. The main clinical suspicion sign for defects in innate immunity, TIR pathway in particular, is recurrent IPD [12, 19]. So in children with recurrent IPD in whom no risk factor has been identified, we strongly recommend a stepwise immune work-up which can begin with the evaluation of antibody responses (since humoral immunodeficiencies are the most common forms of PID representing over 60–70% of all forms), followed by innate immunity evaluation (CH50, Howell-Jolly and, finally, TIR defects, if the above-mentioned are normal).

In conclusion, recurrent IPD in children is a rare condition. Whenever we face it, a predisposing condition needs to be searched for. In the absence of underlying diseases known to predispose to recurrent IPD (which currently indicates pneumococcal vaccination), immunological status of patients should be studied in depth, including TIR pathway, and also followed-up periodically, enabling the implementation of preventive measures, such as pneumococcal vaccination, antibiotic prophylaxis and other specific treatments, if needed. However, if recurrent IPD is a relapse of pneumonia, microbiological factors, rather than immune factors, seem to play a pivotal role.
